# Systemic Lupus Erythematosus Presenting with Alveolar Hemorrhage

**DOI:** 10.1155/2018/8218904

**Published:** 2018-09-16

**Authors:** Omar Tolaymat, Florentina Berianu

**Affiliations:** Mayo Clinic Florida, Department of Rheumatology, 4500 San Pablo Road, Jacksonville, FL 32224, USA

## Abstract

**Introduction:**

Diffuse alveolar hemorrhage is a rare presentation of systemic lupus erythematosus. Early diagnosis and appropriate treatment can improve outcome.

**Case Report:**

An 18-year-old male presented with hemoptysis and respiratory distress requiring orotracheal intubation. Laboratory tests showed positive anti-nuclear antibody and anti-dsDNA and low C3 and C4. Bronchoalveolar lavage became progressively hemorrhagic after each aliquot. He was treated with pulse methylprednisolone, cyclophosphamide, and plasma exchanges.

**Discussion:**

Alveolar hemorrhage is a rare initial presentation of lupus, with mortality rates reported at about 50%. Lupus should be considered in those presenting with alveolar hemorrhage since delay in therapy may cause a rapid deterioration of the patient. The diagnosis of SLE is illusive when DAH is the presenting symptom. Since early diagnosis and appropriate institution of treatment improve outcome, it is important to keep lupus in mind as an etiology of alveolar hemorrhage. Pulse methylprednisolone, cyclophosphamide, and plasmapheresis therapy resulted in rapid improvement of respiratory function in our patient.

## 1. Introduction

Diffuse alveolar hemorrhage (DAH) is a rare complication of systemic lupus erythematosus (SLE). It is however exceedingly unusual as the presenting manifestation of SLE [1]. This paper will discuss a patient presenting with diffuse alveolar hemorrhage (DAH) as the initial symptoms of lupus. Early diagnosis and appropriate treatment can improve outcome. Therefore, it is necessary to consider undiagnosed SLE as a cause of DAH at initial presentation.

## 2. Case Report

An 18-year-old male with no past medical history presented as an outside hospital transfer for acute respiratory failure. Approximately 2 months before, he began to have a cough associated with intermittent hemoptysis. He was evaluated at an urgent care clinic and received antibiotics without resolution of his symptoms. At that time, his renal function and urinalysis were within the normal limits. He denied history of joint pain or subjective joint swelling. Dyspnea progressed, and he was taken to his local hospital. On transfer, patient was intubated and on mechanical ventilation.

On physical examination, he was intubated and sedated. Lungs were with coarse breath sound bilaterally. There was bilateral swelling of his knees.

Laboratory studies showed hemoglobin of 8.4 g/dL, WBC count of 5.6/L with a lymphopenia, platelet count of 126/L, creatinine of 1.6 mg/dL, INR of 1, and PTT of 26.6 seconds. Urinalysis was with 41 red blood cells, and protein to creatinine ratio was 0.94. Anti-nuclear antibody (ANA) was greater than 12, anti-dsDNA was 507 IU/mL, C3 was 63 mg/dL, C4 was 8 mg/dL, and anti-cardiolipin IgM was 15.8 mpl.

His anti-smith,anti-MPO,anti-PR3,anti-cardiolipin IgG, anti-beta 2 glycoproteins IgG/IgM, anti-GBM, lupus anticoagulant, and cryoglobulins were negative.

**Figure 1:**
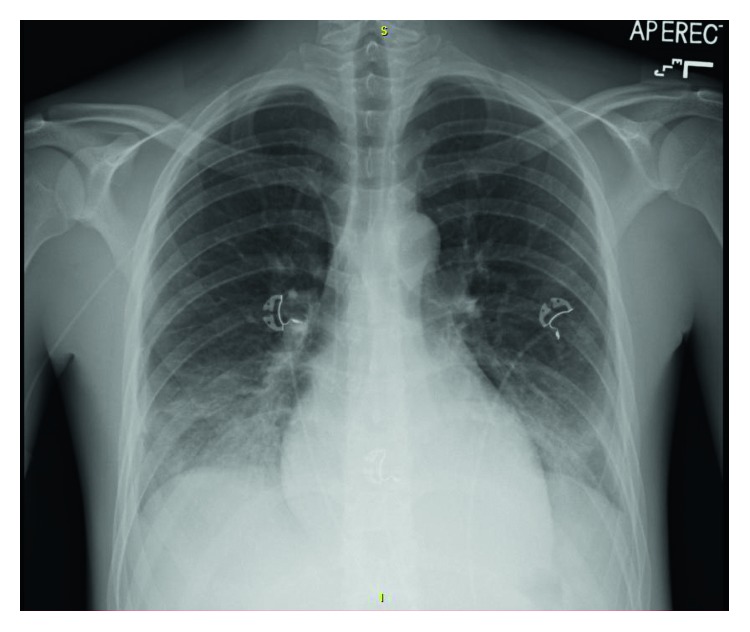
Bibasilar opacities.

**Figure 2:**
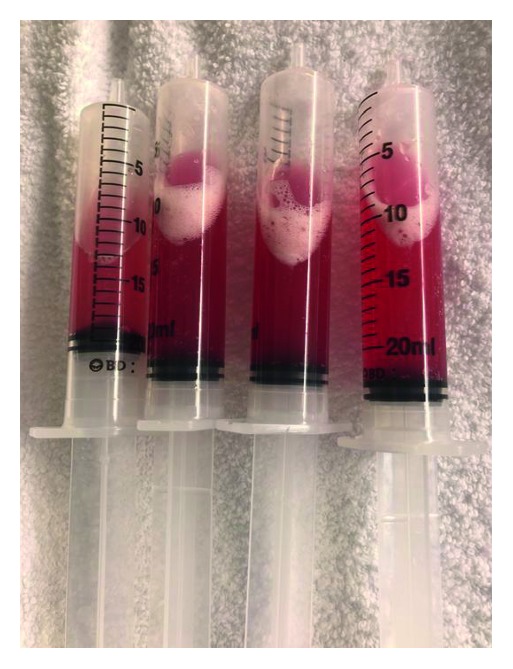
Progressively hemorrhagic aliquots.

Blood and sputum cultures were negative. Chest X-ray showed bibasilar airspace disease (Figure 1). Bronchoalveolar lavage showed progressively hemorrhagic aliquots (Figure 2).

He was treated with pulse dose methylprednisolone for three days, one dose of intravenous cyclophosphamide, and five days of plasmapheresis. Shortly after, he was extubated and weaned to room air. His hemoptysis resolved with stabilization of his hemoglobin. He was discharged in the stable condition.

## 3. Discussion

Classic presentation of diffuse alveolar hemorrhage (DAH) includes dyspnea, cough, and hemoptysis. However, hemoptysis is present in only 44% of cases at the time of presentation [2]. Chest radiographs typically show bilateral central opacities with peripheral sparing. BAL shows progressively hemorrhagic aliquots, which is diagnostic of alveolar hemorrhage.

The differential diagnosis of DAH is broad and includes medications, coagulopathy, illicit drugs, and other autoimmune diseases including systemic vasculitis, good pastures syndrome, and antiphospholipid antibody syndrome.

Our patient meets the Systemic Lupus International Collaborating Clinics (SLICC) classification criteria for lupus with positive ANA, anti-dsDNA, arthritis, nephritis, and low complement.

DAH in SLE is considered life-threatening. Mortality rates have been found to be about 50% [1]. It is also a rare complication and initial manifestation of SLE. In a 10-year retrospective chart review of 510 patients admitted for SLE, there were 19 episodes of DAH in 15 patients. DAH as the initial manifestation of SLE was found in only three of the 510 admissions.

Etiology of DAH in SLE is unclear. In 8 of 10 patients, pulmonary capillaritis was present on biopsy [1].

The most common associated organ involvement in SLE patient with DAH is nephritis (about 70% of cases). Anti-dsDNA was elevated in 75% of cases and low complement in 86% of cases [3].

Despite high mortality rates, there are no randomized clinical trials for appropriate treatment. In a systematic review of 174 episodes of DAH in SLE, corticosteroids were almost universally used. Cyclophosphamide was used in 54% of cases, and plasmapheresis was used in 31% of cases [3].

Few case-control studies have focused on risk factors of DAH in SLE. In a case-control study of 22 SLE and DAH patients, multivariate analysis found thrombocytopenia and low C3 as independent risk factors. Patients with antiphospholipid antibody syndrome (APS) were not excluded in this study [4] Thrombocytopenia is a well-known manifestation of APS which occurs more frequently in patients with APS associated with SLE [5, 6]. Primary APS is thought to be able to cause DAH in the absence of thromboembolic disease [7].

In another case-control study with 21 SLE patients with DAH, multivariate analysis found a high SLE disease activity index score and coexisting neuropsychiatric illness as independent risk factors [8].

A retrospective cohort study of SLE patients with DAH by Dam Kim et al. revealed 47 patients over 10 years with diffuse lung infiltrates. 24 of these patients satisfied the study criteria for DAH. These patients were compared with the 23 patients that did not satisfy the criteria for DAH. Low C4, decreased Hb, and hypoxia were statistically significant [9].

DAH is a rare initial manifestation and deadly complication of SLE. It is important to keep SLE in the differential of patients presenting with DAH because of its high mortality. Early and aggressive therapy should be implemented to improve outcome. The use of pulse dose steroids, plasmapheresis, and cyclophosphamide resulted in improvement in our patient's respiratory status.

## References

[1] Zamora M., Warner M., Ruder R., Schwarz M. (1997). Diffuse alveolar hemorrhage and systemic lupus erythematosus: clinical presentation, histology, survival and outcome. *Medicine*.

[2] Martínez-Martínez M. U., Abud-Mendoza C. (2012). Recurrent diffuse alveolar haemorrhage in a patient with systemic lupus erythematosus: long-term benefit of rituximab. *Lupus*.

[3] Ednalino C., Yip J., Carsons S. E. (2015). Systematic review of diffuse alveolar hemorrhage in systemic lupus erythematosus focus on outcome and therapy. *JCR: Journal of Clinical Rheumatology*.

[4] Kazzaz N. M., Coit P., Lewis E. E., McCune W. J., Sawalha A. H., Knight J. S. (2015). Systemic lupus erythematosus complicated by diffuse alveolar haemorrhage: risk factors, therapy and survival. *Lupus Science and Medicine*.

[5] Uthman I., Godeau B., Taher A., Khamashta M. (2008). The hematologic manifestations of the antiphospholipid syndrome. *Blood Reviews*.

[6] Cervera R., Boffa M. C., Khamashta M. A., Hughes G. R. V. (2009). The Euro-Phospholipid project: epidemiology of the antiphospholipid syndrome in Europe. *Lupus*.

[7] Deane K. D., West S. G. (2005). Antiphospholipid antibodies as a cause of pulmonary capillaritis and diffuse alveolar hemorrhage: a case series and literature review. *Seminars in Arthritis and Rheumatism*.

[8] Kwok S.-K., Moon S.-J., Ju J. (2011). Diffuse alveolar hemorrhage in systemic lupus erythematosus: risk factors and clinical outcome: results from affiliated hospitals of Catholic University of Korea. *Lupus*.

[9] Kim D., Choi J., Cho S. K. (2017). Clinical characteristics and outcomes of diffuse alveolar hemorrhage in patients with systemic lupus erythematosus. *Seminars in Arthritis and Rheumatism*.

